# Reconstructing antibody dynamics to estimate the risk of influenza virus infection

**DOI:** 10.1038/s41467-022-29310-8

**Published:** 2022-03-23

**Authors:** Tim K. Tsang, Ranawaka A. P. M. Perera, Vicky J. Fang, Jessica Y. Wong, Eunice Y. Shiu, Hau Chi So, Dennis K. M. Ip, J. S. Malik Peiris, Gabriel M. Leung, Benjamin J. Cowling, Simon Cauchemez

**Affiliations:** 1 WHO Collaborating Centre for Infectious Disease Epidemiology and Control, School of Public Health, Li Ka Shing Faculty of Medicine, The University of Hong Kong, Hong Kong Special Administrative Region, China; 2 Laboratory of Data Discovery for Health Limited, Hong Kong Science and Technology Park, New Territories, Hong Kong; 3 HKU-Pasteur Research Pole, The University of Hong Kong, Hong Kong Special Administrative Region, China; 4 Mathematical Modelling of Infectious Diseases Unit, Institut Pasteur, UMR2000, CNRS, Paris, France

**Keywords:** Infectious diseases, Epidemiology, Influenza virus, Computational models

## Abstract

For >70 years, a 4-fold or greater rise in antibody titer has been used to confirm influenza virus infections in paired sera, despite recognition that this heuristic can lack sensitivity. Here we analyze with a novel Bayesian model a large cohort of 2353 individuals followed for up to 5 years in Hong Kong to characterize influenza antibody dynamics and develop an algorithm to improve the identification of influenza virus infections. After infection, we estimate that hemagglutination-inhibiting (HAI) titers were boosted by 16-fold on average and subsequently decrease by 14% per year. In six epidemics, the infection risks for adults were 3%–19% while the infection risks for children were 1.6–4.4 times higher than that of younger adults. Every two-fold increase in pre-epidemic HAI titer was associated with 19%–58% protection against infection. Our inferential framework clarifies the contributions of age and pre-epidemic HAI titers to characterize individual infection risk.

## Abstract (web summary)

Serological classification of influenza infection has classically been based on a four-fold or higher increase in antibody levels, but this approach may not be optimal. Here, the authors develop a Bayesian model to improve identification of infections in serological samples by accounting for individual antibody dynamics.

## Introduction

Each year, influenza virus causes an estimated three to five million severe illnesses and 400,000 deaths on average^1^. In addition, avian influenza viruses can occasionally adapt to humans and cause influenza pandemics. A thorough characterization of the risk factors for influenza virus infection is critical to optimize mitigation strategies, and an important component of this is correct identification of infected persons. However, it can be challenging to determine whether a person has experienced an influenza virus infection, since most infections are associated with mild disease or are asymptomatic^2–4^. Serological studies can allow identification of infections regardless of illness severity, and can be used to estimate the incidence of influenza virus infections in different regions and in persons of different ages^2,5–8^.

For more than 70 years, scientists have relied on an ad-hoc rule whereby a 4-fold or greater rise in hemagglutination-inhibiting (HAI) titers in paired sera, which are collected from the same persons before and after an epidemic in longitudinal studies, is considered as evidence of influenza virus infection^9,10^. Although this can capture some asymptomatic and subclinical infections, there are a number of known limitations to this heuristic, such as misclassification due to measurement error^11^, and ‘non-bracketing’ issue in which the first serum is collected after the start of an epidemic so that the paired sera may not neatly bracket the epidemic period^12^. Consequently it can be difficult to estimate infection rates in communities^11,12^, and characterize disease severity^13^, disease burden^1,14^ and risk factors for infection^15^ since all of these require accurate classification of infected vs uninfected persons. Otherwise, other information would be required, such as the sensitivity and specificity of the 4-fold criterion.

Here, we develop an analytical framework to address this challenge. We develop a novel Bayesian model to characterize the dynamics of influenza HAI titers from the analysis of a large cohort of 2353 individuals followed for up to 5 years in Hong Kong. Using this detailed understanding of HAI dynamics, we build an algorithm that can identify influenza infections without having to rely on fixed and arbitrary cut-offs. We use our approach to build a comprehensive picture of the circulation of influenza in Hong Kong and its footprint on HAI titers in the population. We then use the model to perform joint estimation of infection risks in different age groups during six influenza epidemics in Hong Kong from 2009 through to 2013, the risk factors for infection, the degree of boosting and waning in HAI titer after infection, and the measurement errors in HAI titer.

## Results

### Study participants and influenza epidemics

**Fig. 1:**
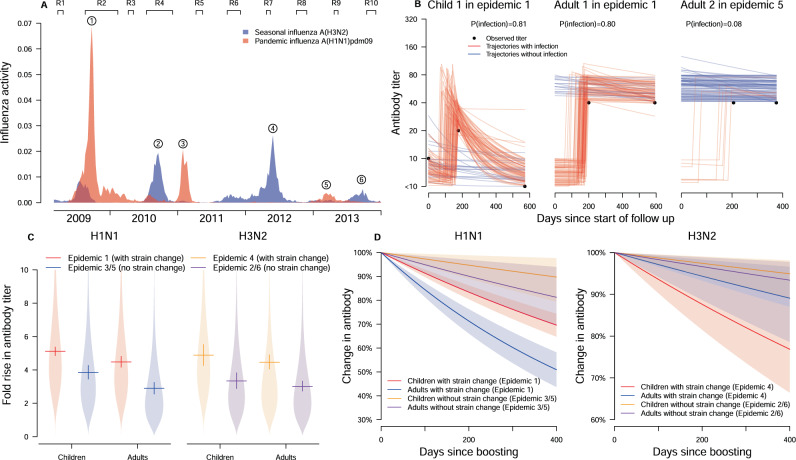
Estimation of individual HAI titer trajectories. **A** Timelines of our study, rounds of blood sample collection, and local influenza activity by surveillance data. Influenza activity for a strain is computed by weekly proportion of outpatients with influenza-like illness multiplied by the weekly proportion of laboratory specimens testing positive for that strain. R1 to R10 indicates the timing of round 1–10 of blood collection. **B** Illustration of HAI titer trajectories, infection status, infection time and pre-epidemic HAI titer if missing in three example individuals. The black dot represents the observed HAI titer, the curve indicates the augmented HAI titer trajectories since the start of an epidemic, red and blue indicate infection and non-infection respectively. Child 1 with 2-fold rise is imputed to be infected in some augmented data to reflect the uncertainty. Adult 1 and 2 with missing pre-epidemic HAI titer, the mid- and post-epidemic HAI titer 3 in log_2_ scale, are from epidemic 1 and 5, respectively. Adult 1 and 2 is estimated to be infected with probability 0.80 and 0.08, respectively. It is because (1) the pre-epidemic titer distribution suggested that adult 3 has a higher pre-epidemic titer than adult 2 (probability of <10 was 0.98 and 0.59 in epidemic 1 and 5, respectively), (2) the infection probability for adults for epidemic 1 (18%) was higher than epidemic 5 (2%). **C** Estimated boosting distribution in HAI titer after infection by subtype. Funnel plots are used to show the distribution. Solid horizontal and vertical lines are used to show the mean and corresponding 95% credible intervals of the estimates based on our estimation approach fitted to data with 2353 individuals. **D** The waning after boosting in HAI titer from infection by subtype. The solid lines are used to show the mean waning of HAI titer trajectories over time and the shadowed areas are used to show the corresponding 95% credible intervals.

A total of 3160 individuals participated in the studies in 2008/09 or 2009/10 including 301 who participated in both studies^16,17^. Participants were then followed up for three additional years^18^ (Supplementary Fig 1). Based on the surveillance data, there were three epidemics of influenza A(H1N1)pdm09 and three seasonal influenza A(H3N2) epidemics (hereafter abbreviated as H1N1 and H3N2 respectively) in the study period (Fig. 1A). For the 6 epidemics, 1321–1851 individuals provided at least one mid-/post-epidemic HAI titer value and were included in the analysis (Supplementary Fig 2, Supplementary Table 1). Figure 1B illustrates titer trajectories for three individuals that are considered uninfected based on the traditional 4-fold rise approach.

### HAI titer dynamics

We estimate that, after infection, geometric mean HAI titers are boosted 3.98 (95% CrI: 3.89, 4.07) log_2_ titers on average, with standard deviation 1.82 (95% CrI: 1.77, 1.88), and 14% (95% CrI: 12%, 16%) of infections are associated with less than 4-fold rises (Fig. 1C). For H1N1, a strain change is associated with a mean boost in adults that was 1.26 log_2_ titers (95% CrI: 0.78, 1.73) higher in epidemic 1 (with strain change; Supplementary Table 3) than in epidemics 3 and 5 (without strain change; Supplementary Table 3). The effect is similar in children (1.58 log_2_ titers; 95% CrI: 1.10, 2.07). For H3N2, the mean boost in epidemic 4 (with strain change; Supplementary Table 3) is also higher than in epidemics 2/6 (without strain change; Supplementary Table 3): 1.54 log_2_ titers (95% CrI: 0.73, 2.39) higher for children and 1.45 log_2_ titers (95% CrI: 0.94, 1.96) higher for adults. For H1N1, the mean boosting in children is 0.64 log_2_ titers (95% CrI: 0.21, 1.04) and 0.95 log_2_ titers (95% CrI: 0.41, 1.50) higher than in adults, in epidemic 1 and epidemic 3/5, respectively. There is no difference in boosting in children and adults for H3N2.

After the boost following infection, the HAI titer starts to wane, with a mean waning rate of 14% (95% CrI: 12%, 15%) a year after infection, and a standard deviation of 22% (95% CrI: 21%, 23%). We also observe substantial differences in the waning rate depending on the epidemic (Fig. 1D). For H1N1, we estimate that the waning in epidemic 1 (with strain change) is 27% (95% CrI: 11%, 40%) and 53% (95% CrI: 27%, 84%) more than in epidemic 3/5 (without strain change) for children and adults respectively. For H3N2, we estimate that the waning in epidemic 4 (with strain change) is 21% (95% CrI: 8%, 40%) larger than in epidemic 2/4 (without strain change) for children, but no difference for adults. Overall, we find that when the circulating strain is substantially different than the previous ones, HAI titers exhibit larger boost and waning than those seen in other years.

We estimate that the 1-sided probability of a 2-fold error is 2.8% (95% CrI: 2.3%, 3.6%) and 5.5% (95% CrI: 4.9%, 6.1%) for H1N1 and H3N2, respectively. The probability that the measurement is erroneous and that observed HAI titer level is a random value from <10 to 5120 is 3.3% (95% CrI: 2.9%, 3.7%).

Our framework allows us to reconstruct the antibody titer dynamics to identify infections probabilistically, integrating information on observed titers, the boosting and waning distribution, measurement error, and influenza activity. For example, our model suggests that the Child 1 in Fig. 1B that has a 2-fold rise in epidemic 1 was likely infected with probability 0.81. Due to irregular seasonality in Hong Kong and unpredictable timing of influenza circulation^12,19–21^, the pre-epidemic titer may be missing and infection may have occurred before the collection of the first sample (Supplementary Fig 2). For example, Adult 1 in Fig. 1B has HAI titer equal to 3 in log_2_ scale in the first and second sample but we infer that this individual has an 80% chance to have been infected. This is because almost all individuals have an HAI titer <10 before the pandemic while there was also high prevalence of infection in the community during the pandemic (estimates shown in the next section). In contrast, Adult 2 in Fig. 1B has the same observed titer pattern in a seasonal epidemic but since the probability of infection in the community is low during this epidemic, it is unlikely this individual was infected (probability of 0.08). These simple examples illustrate the additional insight one can gain from an analysis that goes beyond the 4-fold criterion and can integrate additional contextual information.

Using the same technique for each individual, among 9463 person-epidemic investigated over six epidemics, we probabilistically identify 1731 infections (95% credible interval (CrI): 1657, 1791). Among these, 45% of those infections (779; 95% CrI: 736, 807) have no pre-epidemic titers and could therefore not be identified by the traditional 4-fold criterion. Among 662 individuals with 2-fold rise in paired sera, we estimate that 24% of those (160; 95% CrI: 139, 192) were infected.

### Infection risk in epidemics

**Fig. 2:**
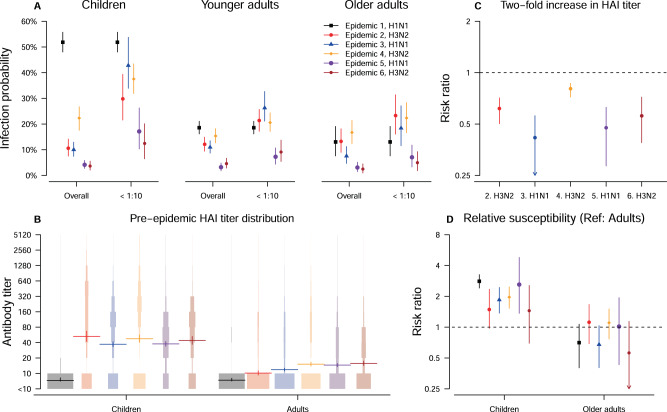
Infection probability and its determinant among six epidemics. **A** Estimates of infection probabilities for children, younger adults and older adults in the six epidemics in our study period. The overall infection probability and infection probability for individuals with pre-epidemic titer of <10 are plotted to show the protective effect of pre-epidemic titers. Points and vertical lines are used to show the mean and corresponding 95% credible intervals of the estimates based on our estimation approach fitted to data with 2353 individuals. **B** The protection associated with 2-fold increase in HAI titers in epidemic 2–6. Points and vertical lines are used to show the mean and corresponding 95% credible intervals of the estimates based on our estimation approach fitted to data with 2353 individuals. **C** The pre-epidemic HAI titer distribution for children and adults in the six epidemics. Funnel plots are used to show the distribution. Points and vertical lines are used to show the mean and corresponding 95% credible intervals of the estimates based on our estimation approach fitted to data with 2353 individuals. **D** Model estimate of the age-relative susceptibility for six epidemics. Points and vertical lines are used to show the mean and corresponding 95% credible intervals of the estimates based on our estimation approach fitted to data with 2353 individuals.

We estimate the infection probability for children (age < 18), adults (age 18–50) and older adults (age > 50) during six epidemics (Fig. 2A). For the 2009 H1N1 pandemic, these probabilities were 52% (95% CrI: 48%, 56%), 19% (95% CrI: 16%, 21%) and 13% (95% CrI: 8%, 19%), respectively. During seasonal epidemics 2–6, they were in the range 4%–22% for children, 3%–15% for adults, and 2%–17% for older adults. However, these overall infection probabilities reflect the combination of the effects of age, the distribution of pre-epidemic HAI titers across age groups and the level of protection associated with different HAI titers. First, the infection probability for individuals with a given pre-epidemic titer could vary by epidemic. The infection probabilities during epidemics 5–6 were smaller than for epidemics 1–4 for individuals with pre-epidemic HAI titer <10, and children were at higher risk than adults even when they both had HAI titers <10 (Fig. 2A). Second, the average pre-epidemic titers for children (log_2_ titer: 3.1) are higher than adults (log_2_ titer: 1.4) among the five seasonal epidemics (Fig. 2B). Third, the protection associated with a two-fold increase in pre-epidemic titer (Fig. 2C) ranges from 38% to 58%, except in epidemic 4, where the protection is only 19% (95% CrI: 13%, 28%). Therefore, after adjusting for the higher average pre-epidemic titers of children, their infection probabilities are still estimated to be 1.6 to 4.4 times higher than that of younger adults in the six epidemics (Fig. 2D).

### Association between infection status in different epidemics

**Fig. 3:**
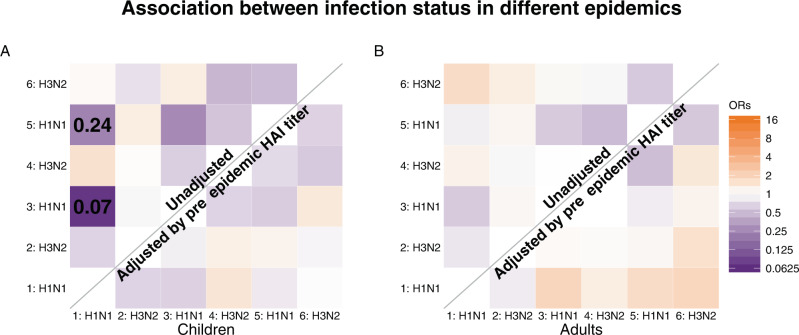
The association between infection status in different epidemics. The association between the infection status in each pair of epidemics in the study period. Upper and lower diagonal shows the odds ratio without adjustment and adjusting pre-epidemic HAI titer respectively. **A**, **B** Indicate the association for children and adults respectively.

We explore the association between infection status in different epidemics, considering individuals that participated in more than one epidemic in our study (*n* = 1853; Supplementary Table 2). For adults, there is no evidence for heterosubtypic protection from previous infections (Fig. 3 and Supplementary Fig 3). For children, infection during H1N1 epidemic 1 provides 93% (95% CrI: 83%, 98%) and 76% (95% CrI: 25%, 96%) protection in H1N1 epidemic 3 and 5, respectively. Such homosubtypic protection is not observed for H3N2. However, the association is no longer significant after adjusting for pre-epidemic HAI titers in the logistic regression, suggesting such protection is captured by information on pre-epidemic HAI titers boosted by previous infections.

### Sensitivity and specificity of identifying infections

We conduct a simulation study to compare the sensitivity and specificity of identifying infections by using our approach or using a 4-fold criterion. We use our model with the model parameters randomly drawn from their posterior distribution to simulate 50 epidemics. When pre-epidemic titers are available, the sensitivity and specificity of our approach are 87% (95% posterior predictive intervals (PPI): 85%, 89%) and 98% (95% PPI: 97%, 98%), which is better than the ones obtained using a 4-fold cutoff point, with 82% (95% PPI: 80%, 84%) sensitivity and 96% (95% PPI: 96%, 97%) specificity. Moreover, our framework performs substantially better when the pre-epidemic titers are unavailable, with 78% (95% PPI: 73%, 81%) sensitivity and 96% (95% PPI: 95%, 97%) specificity compared to the 4-fold rise case definition. Using a 4-fold criterion would have only 22% (95% PPI: 20%, 24%) sensitivity and 98% (95% PPI: 98%, 99%) specificity, due to the ‘non-bracketing’ problem that individuals infected before the collection of the first serum samples are misclassified as uninfected ^12^.

### Model validation and adequacy

**Fig. 4:**
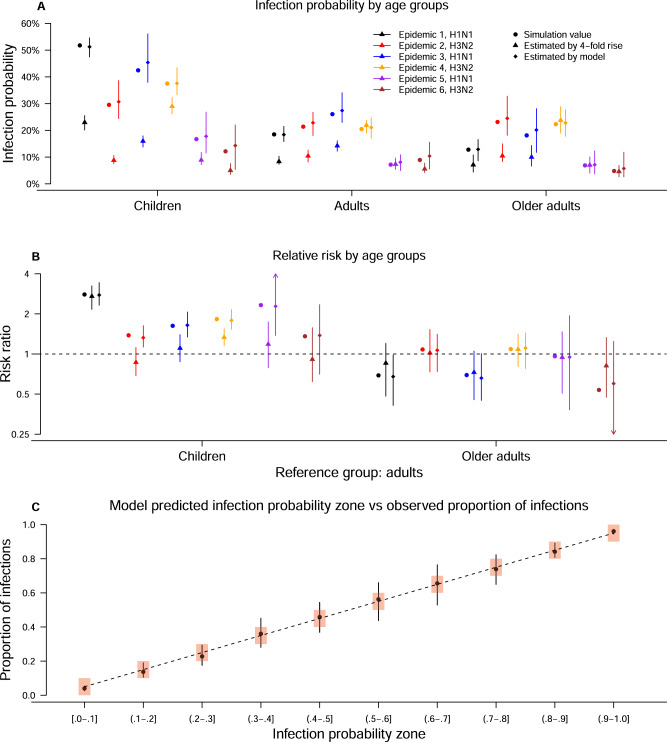
Comparison of using 4-fold criterion and using model estimates to estimate infection probabilities and relative susceptibility among age groups by simulation. **A**, **B** Show the infection probabilities and relative susceptibility among age groups estimated by a 4-fold criterion and by our proposed approach respectively. Points and vertical lines are used to show the mean, 2.5% and 97.5% percentile of the estimates from 50 simulated epidemics based on our data with 2353 individuals. **C** The proportion of infected individuals in the groups with different infection probability zone (red rectangles) estimated by our inference from simulation study. Points and vertical lines are used to show the mean, 2.5% and 97.5% percentile of the estimates from 50 simulated epidemics based on our data with 2353 individuals.

Based on 50 simulated epidemics, we found that the 4-fold criterion leads to an underestimation by 44–70% of infection probabilities for the first three epidemics in which there are non-bracketing issue so that the pre-epidemic titer for some individuals are unavailable (Fig. 4A). In addition, the infection probabilities for children are underestimated by 23–59% for the other three epidemics with no non-bracketing issue. Furthermore, the age relative susceptibility may be biased if using a 4-fold criterion to identify infections, particularly for children (Fig. 4B).

In a simulation study with 50 epidemics, we validate that our approach can accurately identify infections by computing the proportion of infections in individuals with model-predicted infection probabilities that fell in the infection probability window (10 intervals from 0 to 1). The proportion of infections are equal to the middle value of each probability window, suggesting that our inference can correctly identify infections (Fig. 4C). Furthermore, the distributions of titer change predicted by the model for children and adults in each epidemic are consistent with the observed ones (among 1000 simulated epidemics) (Supplementary Fig 4). In 50 simulated epidemics, we find our approach could adequately estimate model parameters with no systematic bias (Supplementary Table 4).

## Discussion

In this study, we developed a Bayesian modeling approach to jointly estimate individual antibody dynamics, identify influenza virus infections, and determine the risk factors for infection. We found that our approach outperformed the use of the 4-fold criterion which may cause under-detection of infections and hence underestimation of infection risks^11,12^. Compared with previous approaches that used the antibody titer response after PCR-confirmed infections to identify infections without using the traditional 4-fold rise approach^22,23^, our approach jointly characterized antibody titer dynamics to identify infections based on serology data alone and, did not require any PCR-related data. Instead, we used surveillance data to inform estimation of infection time, which would be much easier to obtain and would be much more suitable for serologic studies ^6^.

We estimated the degree of boosting of HAI titers after H1N1 and H3N2 infections, and their waning after the boost. We estimated that higher boosting occurred in children, and after strain changes in epidemics, which is consistent with the results from previous studies^24,25^. Both associations are likely caused by the infection histories, as previous exposure to similar strains would reduce the HAI titer response^26–28^ and children are less likely to have been previously exposed than adults. We found that the waning in HAI titer was higher in epidemics with strain change for both H1N1 and H3N2. Such higher waning was also observed in other studies^7,12,23,29–31^. Further studies are required as the waning rate could depend on other factors, such as the definition of cases, previous exposure or age ^12,22,26^.

Our findings indicate that there is a complex interaction between age and individual immunity. In our framework that accounted for variability in HAI assays^32^, we find that the protection associated with HAI titer could vary by epidemic, similar to other studies^15,19,30,33–35^. Also, we find that the infection probabilities for children and adults could differ after adjusting for HAI titers, which could be because HAI titers only capture part of the immunity for adults^22,26,34^, or that children have greater exposure to infection for various reasons^33,36^. Despite these age differences in infection probabilities, we still find that HAI titers predict infection probabilities at the individual level, and the protection from prior infection could be explained by HAI titer that was boosted by previous infections. This suggests that the distribution of HAI titers in a population could be a useful measure of the proportion of individuals with protection, as a measure of population immunity ^7^.

Our approach consistently outperforms the traditional 4-fold criterion for both sensitivity and specificity. Therefore, our approach could give a more accurate estimate of the incidence of infections and hence more accurate determination of risk factors and disease burden. In particular, our approach could account for the different boosting and waning patterns and therefore would be generalizable to populations with persons with different boosting and waning distribution for different influenza virus strains or even other pathogens with similar patterns after infections^37^. Furthermore, our approach could still perform well when there is a “non-bracketing” issue that collected sera may not neatly bracket the epidemics of interest. Such performance would be critical as “non-bracketing” is unavoidable in tropical and subtropical regions, or in unpredictable influenza pandemics ^20,38,39^.

Our study has some limitations. We used a proxy measure of influenza activity in the community (Fig. 1A) and the reliability of estimates would depend on the accuracy of the proxy to reflect the relative infection risk over time. Although the proxy is not age-specific, our previous study suggested that the incidence rate of H1N1 infection in 2009 pandemic were similar among different age groups^40^. Since the rise in HAI titer from vaccination is indistinguishable from natural infections, we excluded all recently vaccinated individuals in this analysis, and our results may not be generalizable if vaccination has an effect on infection risk over more than one epidemic.

In conclusion, here we introduced a new approach to jointly characterize antibody dynamics and identify influenza virus infections from serological data that addresses key limitations of the tradition 4-fold criterion. Within our inferential framework, we were able to clarify the contributions of age and pre-epidemic titers to characterize individual infection risks.

## Methods

### Study design

Data were collected from two community-based randomized controlled trials (RCTs) for evaluating direct and indirect benefits of influenza vaccination (Supplementary Note 1)^16,17^. In the RCTs conducted in 2008/09 and 2009/10, 119 and 796 households were recruited. Serum specimens were collected at the start of the study, and after 6 and 12 months from all participants. In the subsequent observational follow-up of the same cohort participants from late 2010 to late 2013 without intervention^18^, serum specimens were collected from all participants in each autumn (October to December), and also each spring (April to May). Receipt of influenza vaccine outside of the trial was recorded annually.

### Ethics

All participants aged 18 years and older gave written informed consent. Proxy written consent from parents or legal guardians was obtained for participants, with additional written assent from those aged 8–17 years. The study protocol was approved by the Institutional Review Board of the University of Hong Kong and by the Hong Kong Department of Health Ethics Committee (Clinical Trials Registration. NCT00792051).

### Laboratory methods

All serum specimens were tested in parallel for antibody responses by hemagglutination inhibition (HAI) assays in serial doubling dilutions from an initial dilution of 1:10 using standard methods^41^. The reciprocal of the highest dilution of serum that prevents complete hemagglutination wells was regarded as the antibody titer. In the pilot (2008/09), serum specimens were tested against A/California/7/2009(H1N1) and A/Brisbane/10/2007(H3N2). In the main trial and second year of follow-up, i.e., 2009/10 and 2010/11, serum specimens were tested against A/California/7/2009(H1N1) and A/Perth/16/2009-like (H3N2). In 2011/12 and 2012/13 serum specimens were tested against A/California/7/2009(H1N1) and A/Victoria/361/2011-like (H3N2). Sera from consecutive years were tested in parallel due to the recommended practice of examining paired sera for evidence of influenza virus infections.

### Surveillance data

Influenza activity in the general community is monitored through a sentinel surveillance network in outpatient clinics, which report the proportion of patients with influenza-like illness defined as a fever >37.8 °C plus a cough or sore throat. The public health laboratory also collects data on the weekly proportion of specimens from sentinel outpatient clinics and local hospitals that tested positive for influenza virus. An incidence proxy is constructed to measure the weekly incidence rate of influenza virus infections in the community, derived as the weekly proportion of outpatients with influenza-like illness multiplied by the weekly proportion of laboratory specimens testing positive for H1N1pdm09 or H3N2 virus^42^. This particular proxy is showed to be a good indication of incidence of H1N1pdm09 virus infection in the community based on hospital admissions ^40^.

### Statistical models

There were at most 13 serum specimens per participant. However, there was no H1N1 and H3N2 activity between November-December 2008 and April 2009. Therefore, serum collection in April 2009 is considered as the first round in our analysis. Post-vaccination sera are excluded in the analysis since they were only available for a subset of children in the 2009/2010 study.

We first identify influenza A epidemics during our study period based on incidence proxy, constructed based on local influenza surveillance data. Then, we identify relevant consecutive titers to estimate the infection risk. For each epidemic, the most recent round of serum collection prior to that epidemic is used to obtain the pre-epidemic titers, to address the non-bracketing issue^12^. Then, any titer that is collected in the next two consecutive rounds (mid-epidemic and post-epidemic titers) are used to infer infection status. Further titers are ignored due to the low sensitivity to detect infections with waning in titers over time^12^. For each epidemic, only unvaccinated participants with mid- or post-epidemic titers are included in the analysis of that epidemic, since interpretation of serology in vaccinated persons can be challenging ^43^.

Titers of 10, 20, 40 … 2560 are translated on a log_2_ scale to 1, 2, …, 9. Undetectable titers <10) are set to 0 on the same scale. While infection has traditionally been defined by 4-fold or greater rise in titers in consecutive pairs of sera, this definition is shown to be suboptimal for estimating infection risk, since some 2-fold rises may also indicate infections with smaller rises^11^. To improve this, we developed a 5-level hierarchical model to reconstruct the infection status, infection time and the unobserved HAI titer trajectory for each individual by describing the infection risk, boosting in antibody after infection and waning in antibody by integrating serology data and surveillance data (Supplementary Note 2). Parameters and latent variables in the model were summarized in Supplementary Table 5. Hence, we could identify infections without using a cutoff of the rise of titers to define infections in our analyses.

The first level of our model was the ‘measurement model’ (Supplementary Note 2). We modeled the underlying titer on a continuous scale, so that a ‘true’ titer between any two dilutions was measured as the lower of the two dilutions when there was no measurement error. For example, a “true” titer of 1.3 is measured as 1. We consider a ‘true’ titer was the underlying but unmeasured titer on a continuous scale, and a ‘measured titer’ is the value that is actually measured by the assay. We use the approach in Cauchemez et al. to model the probability of 2-fold error on the left or right side ^11^.

The second level of the model was the HAI titer dynamics model (Supplementary Note 2). The magnitude of the boosting and the waning was Gamma distributed, and their characterizing parameters are estimated in the inference. We allow these distributions to be different for children and adults, for epidemics with or without strain change compared with previous epidemic of same subtype (H1N1 or H3N2), and by subtype.

The third level of the model was the infection model (Supplementary Note 2), that describes the daily infection risk during epidemics. In agreement with our previous analysis^12^, we assume that the scaling parameter changed on November 21, 2009 to account for the fact that the sentinel surveillance system is affected by the 2009 pandemic H1N1 outbreak^44^. The pre-epidemic titer and age groups were considered as covariates of infection risk in the model. The fourth level of the model for the distribution of pre-epidemic titer (Supplementary Note 2). The fifth level of the model specifies our priors on model parameters.

### Model Inference

To infer the unobserved HAI titer trajectories, we use a Bayesian data augmentation framework^45^. We developed a reversible-jump Markov Chain Monte Carlo (MCMC) algorithm to explore the joint distribution of model parameters and latent variables (Supplementary Note 3). We updated model parameters with a Metropolis-Hastings algorithm. Infection times, individual boosting and waning parameters, and true titers for each individual were jointly updated with a Metropolis-Hastings algorithm conditional on their augmented infection status. Finally, we used a reversible-jump MCMC approach to add or remove infections, informed by the pattern of HAI titers, and also the infection risk of a particular epidemic. Statistical analyses were conducted using R version 4.0.5 (R Foundation for Statistical Computing, Vienna, Austria).

### Association between infection status in different epidemics

We utilize the longitudinal feature of our study to explore association between infection status in different epidemics (Supplementary Note 4). To estimate the association, we randomly select 100 augmented infection status in the inference to reconstructed datasets. For each reconstructed dataset, we conduct logistic regression for infection status for each pair of epidemics in our study. Hence, we could estimate the homosubtypic or heterosubtypic protection from previous infections, defined as one minus odds ratio, based on the subtypes of the pair of epidemics (Supplementary Table 4). We further include the pre-epidemic HAI titer in the logistic regression, to determine if any identified protection could be explained by pre-epidemic HAI titer. We use a bootstrap approach to account for sampling uncertainty for the above analyses ^37^.

### Model validation and adequacy

A simulation study is conducted to confirm that our algorithm could provide unbiased estimates of model parameters. In each simulation, a dataset is simulated with parameters equal to their posterior mean, and with a structure identical to the observed dataset, including the availability of titer measurement, infection status and age (Supplementary Note 5). We also track the sensitivity and specificity for using our proposed model and 4-fold criterion to identify infections, and track the infection probabilities by age groups, and relative susceptibility among age groups computed by using a 4-fold criterion. In our model, we defined infection in an individual as the estimated probability of infection for that individual being >0.5, in computing sensitivity and specificity. When applying the 4-fold criterion, we assumed that the baseline HAI titer for individuals without a baseline sample were missing, so that they were assumed to be not infected before the collection of the first serum, which could be either a mid-season or post-season sample. We check the model adequacy by comparing the distribution of titer changes for each age group and each epidemic in observed data and the 1000 simulated epidemics.

### Reporting summary

Further information on research design is available in the Nature Research Reporting Summary linked to this article.

## Supplementary information

Supplementary Information

Reporting Summary

## Data Availability

All the data used in the analysis is available at Github: https://github.com/timktsang/influenza_titer_reconstruction.

## Code availability

Statistical analyses were conducted using R version 4.0.5 (R Foundation for Statistical Computing, Vienna, Austria). Code is available at Github: https://github.com/timktsang/influenza_titer_reconstruction.
