# Author Correction: Reconstructing antibody dynamics to estimate the risk of influenza virus infection

**DOI:** 10.1038/s41467-023-36822-4

**Published:** 2023-02-23

**Authors:** Tim K. Tsang, Ranawaka A. P. M. Perera, Vicky J. Fang, Jessica Y. Wong, Eunice Y. Shiu, Hau Chi So, Dennis K. M. Ip, J. S. Malik Peiris, Gabriel M. Leung, Benjamin J. Cowling, Simon Cauchemez

**Affiliations:** 1 WHO Collaborating Centre for Infectious Disease Epidemiology and Control, School of Public Health, Li Ka Shing Faculty of Medicine, The University of Hong Kong, Hong Kong Special Administrative Region, China; 2 Laboratory of Data Discovery for Health Limited, Hong Kong Science and Technology Park, New Territories, Hong Kong; 3 HKU-Pasteur Research Pole, The University of Hong Kong, Hong Kong Special Administrative Region, China; 4 Mathematical Modelling of Infectious Diseases Unit, Institut Pasteur, UMR2000, CNRS, Paris, France

**Keywords:** Infectious diseases, Epidemiology, Influenza virus, Computational models

Correction to: *Nature Communications* 10.1038/s41467-022-29310-8, published online 23 March 2022

In this article the funding from ‘European Union’s Horizon 2020 research and innovation program under VEO grant agreement No. 874735’ was omitted. The original article has been corrected.

