# Supplementary material for: Reconstructing antibody dynamics to estimate the risk of influenza virus infection
Source: Nat Commun. 2022 Mar 23;13:1557. doi: 10.1038/s41467-022-29310-8 (PMC8943152; doi:10.1038/s41467-022-29310-8)
Supplement: Supplementary file 1 — Supplementary Information [file 41467_2022_29310_MOESM1_ESM.pdf]

Supplementary Material

for 'Reconstruction of antibody dynamics to estimate the risk of influenza virus infection'

Tim K. Tsang[1,2], Ranawaka A. P. M. Perera[1,3], Vicky J. Fang[1], Jessica Y. Wong[1], Eunice Y. Shiu[1], Hau Chi So[1], Dennis K. M. Ip[1], J. S. Malik Peiris[1,3], Gabriel M. Leung[1,2], Benjamin J. Cowling[1,2*], Simon Cauchemez[4*]

* These authors jointly supervised this work

Affiliations:

1. WHO Collaborating Centre for Infectious Disease Epidemiology and Control, School of Public Health, Li Ka Shing Faculty of Medicine, The University of Hong Kong, Hong Kong Special Administrative Region, China.

2. Laboratory of Data Discovery for Health Limited, Hong Kong Science and Technology Park, New Territories, Hong Kong.

3. HKU-Pasteur Research Pole, The University of Hong Kong, Hong Kong Special Administrative Region, China.

4. Mathematical Modelling of Infectious Diseases Unit, Institut Pasteur, UMR2000, CNRS, Paris, France.

Corresponding author: Prof. Benjamin J Cowling, School of Public Health, The University of Hong Kong, 21 Sassoon Road, Pokfulam, Hong Kong.

Tel: +852 3917 6711; Fax: +852 3520 1945; email: bcowling@hku.hk

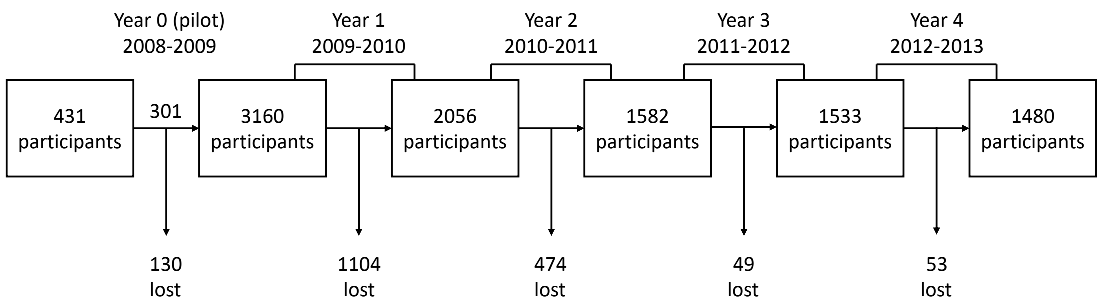

Supplementary Figure 1. The flow diagram of the recruitment and follow-up of the two trails, generated the data for our analysis.

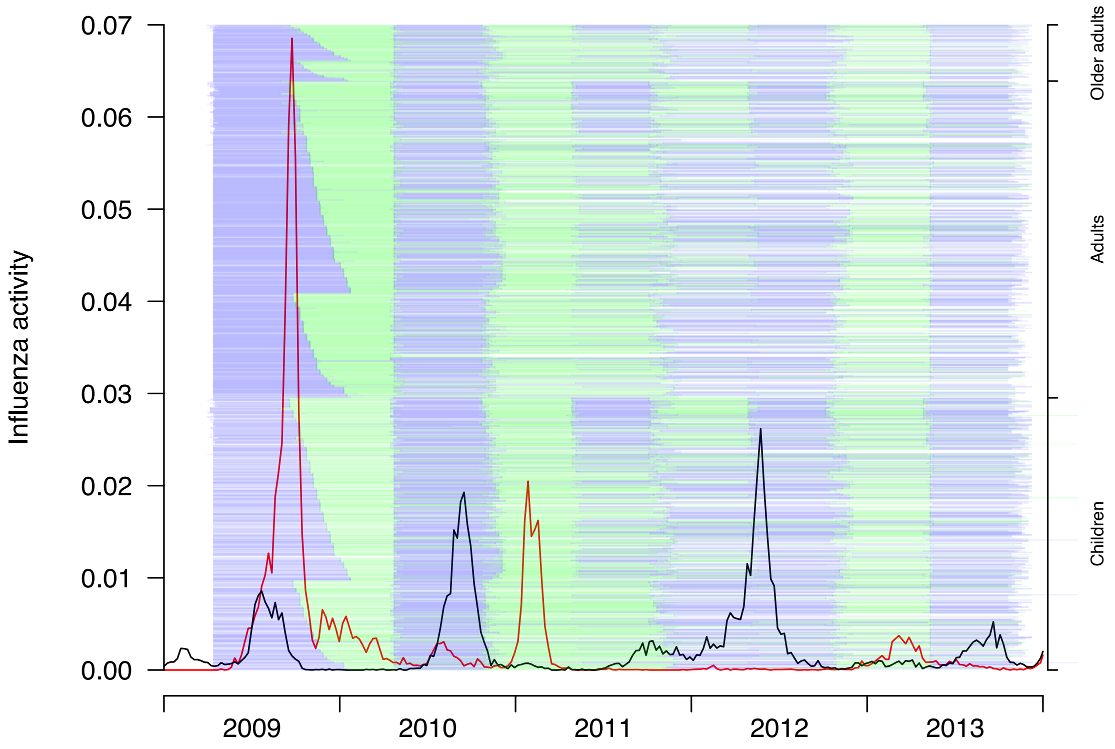

Supplementary Figure 2. A timeline of the study and local influenza activity for H1N1 and H3N2 epidemic. Red and black lines indicate the influenza activity for H1N1 and H3N2 respectively. Purple and green lines indicate sera collected in half-year intervals. Gray lines indicate sera collected in one-year intervals.

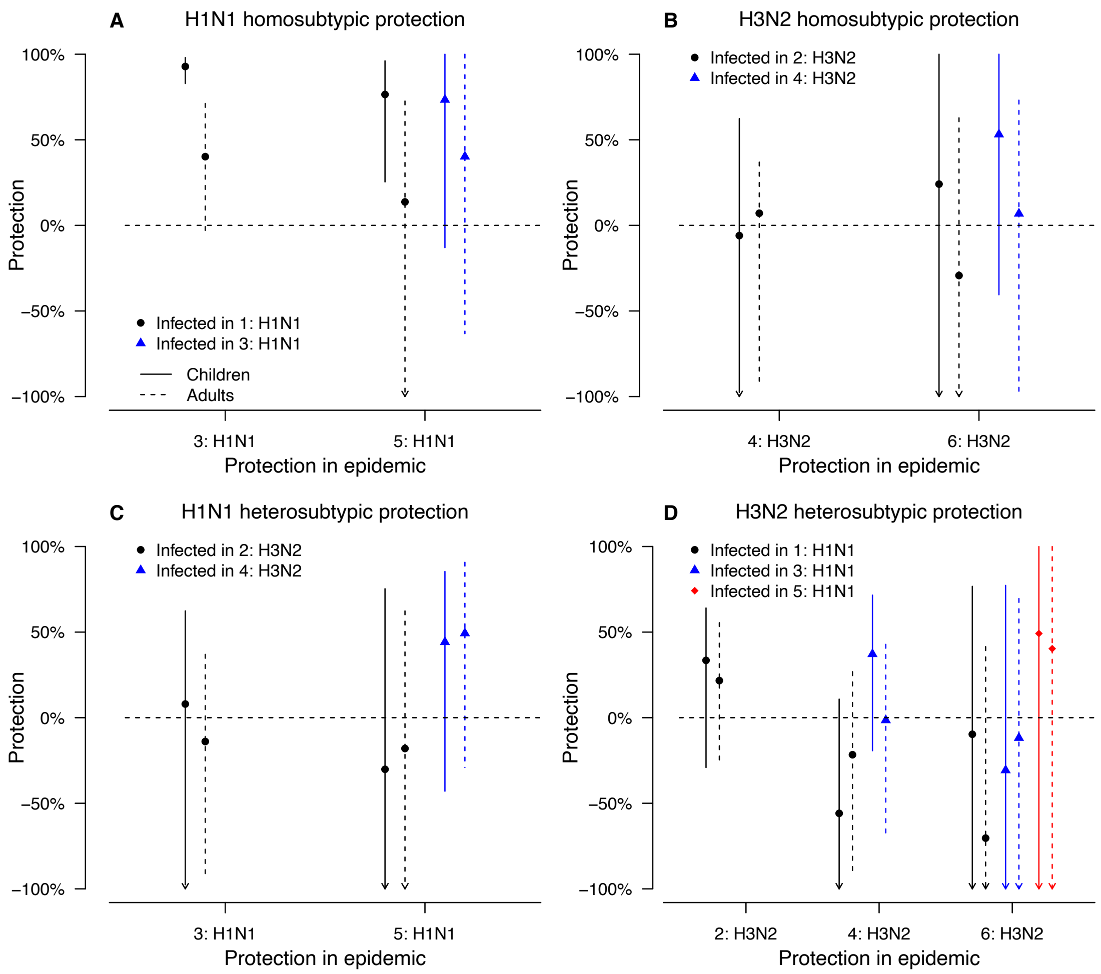

Supplementary Figure 3. The protection associated with infection in previous epidemics estimated by logistic regression. Homosubtypic and heterosubtypic protection was defined in Table S1. Panel A to D was homosubtypic protection against H1N1 and H3N2 infection, heterosubtypic protection against H1N1 and H3N2 infection respectively. Points and vertical lines are used to show the mean and corresponding 95% credible intervals of the estimates based on our estimation approach fitted to data with 2353 individuals.

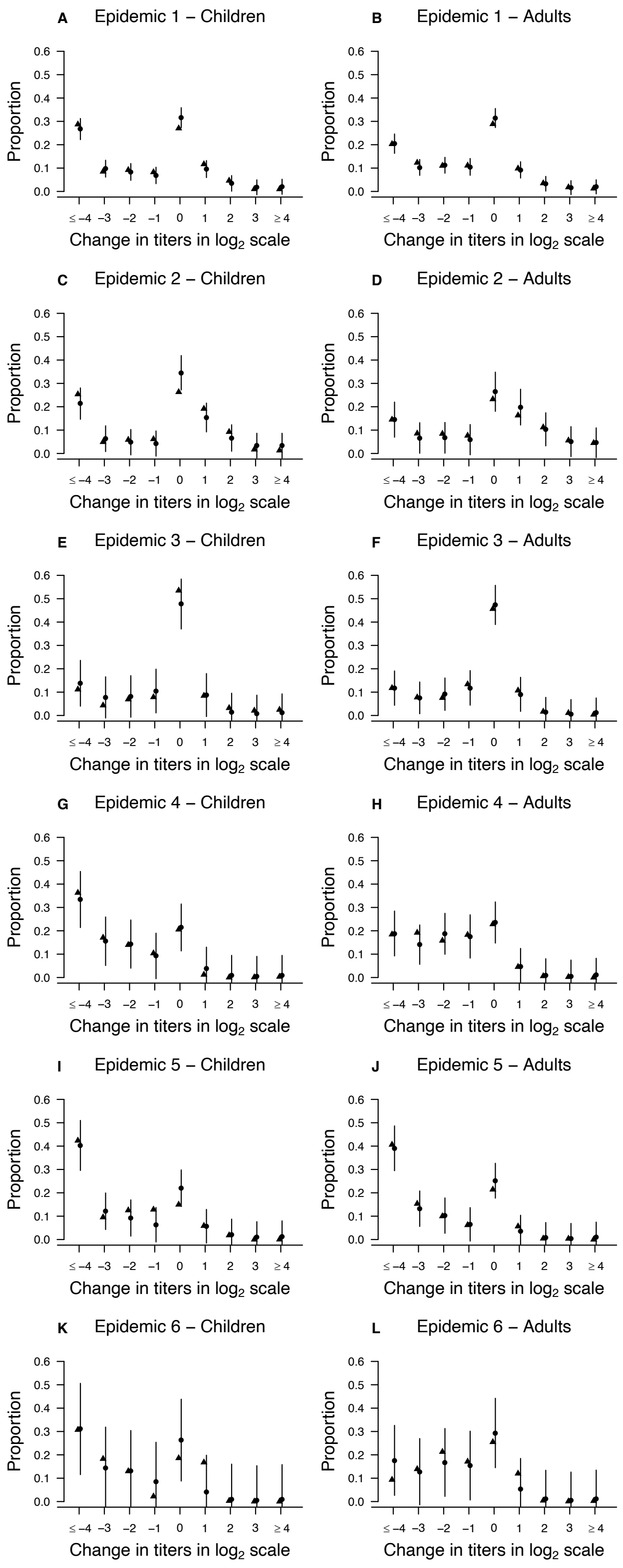

Supplementary Figure 4. The observed and model-simulated distribution of titer changes among six epidemics for children and adults. Panel A and B are the distribution for children and adults in epidemic 1 respectively. Panel C and D, Panel E and F, Panel G and H, Panel I and J , Panel K and L are for epidemic 2, 3, 4, 5 and 6 respectively. Points

and vertical lines are used to show the mean and corresponding 95% credible intervals of the estimates based on our estimation approach fitted to data with 2353 individuals.

Supplementary Table 1. Availability of serum samples for individuals among epidemics.

Epidemics	1: H1N1	2: H3N2	3: H1N1	4: H3N2	5: H1N1	6: H3N2
Total	1851	1781	1753	1427	1330	1321
Children						
Total	612 (33%)	812 (46%)	736 (42%)	578 (41%)	501 (38%)	459 (35%)
With pre-epidemic titer	80 (13%)	326 (40%)	730 (99%)	571 (99%)	495 (99%)	224 (49%)
Without pre-epidemic titer	532 (87%)	486 (60%)	6 (1%)	7 (1%)	6 (1%)	235 (51%)
Adults						
Total	1056 (57%)	778 (44%)	787 (45%)	622 (44%)	572 (43%)	558 (42%)
With pre-epidemic titer	113 (11%)	322 (41%)	781 (99%)	613 (99%)	552 (97%)	268 (48%)
Without pre-epidemic titer	943 (89%)	456 (59%)	6 (1%)	9 (1%)	20 (3%)	290 (52%)
Older adults						
Total	183 (10%)	191 (11%)	230 (13%)	227 (16%)	257 (19%)	304 (23%)
With pre-epidemic titer	17 (9%)	80 (42%)	229 (100%)	224 (99%)	251 (98%)	152 (50%)
Without pre-epidemic titer	166 (91%)	111 (58%)	1 (0%)	3 (1%)	6 (2%)	152 (50%)

Supplementary Table 2: Classification of homosubtypic and heterosubtypic protection

Infection risk in epidemic	Homosubtypic protection	Heterosubtypic protection
1: H1N1		
2: H3N2		Previous infection in 1: H1N1 vs no previous H1N1 infection
3: H1N1	Previous infection in 1: H1N1 vs no previous H1N1 infection	Previous infection in 2: H3N2 vs no previous H3N2 infection
4: H3N2	Previous infection in 2: H3N2 no previous H3N2 infection	Previous infection in 1: H1N1 or 3: H1N1 vs no previous H1N1 infection
5: H1N1	Previous infection in 1: H1N1 or 3: H1N1vs no previous H1N1 infection	Previous infection in 2: H3N2 or 4: H3N2 vs no previous H3N2 infection
6: H3N2	Previous infection in 2: H3N2 or 4: H3N2 vs no previous H3N2 infection	Previous infection in 1: H1N1 or 3: H1N1 or 5: H1N1 no previous H1N1 infection

Supplementary Table 3. Mean boost after infections by influenza type.

	Children	Adults	Difference in mean boost by age groups
H1N1			
With strain change (Epidemic 1)	5.11 (4.86, 5.37)	4.48 (4.16, 4.81)	0.64 (0.21, 1.04)
Without strain change (Epidemic 3 and 5)	3.85 (3.45, 4.27)	2.9 (2.56, 3.25)	0.95 (0.41, 1.5)
Difference in mean boost by with or without strain change	1.27 (0.78, 1.73)	1.58 (1.1, 2.07)	
H3N2			
With strain change (Epidemic 1)	4.89 (4.26, 5.52)	4.46 (4.06, 4.9)	0.43 (-0.31, 1.13)
Without strain change (Epidemic 3 and 5)	3.34 (2.88, 3.83)	3.02 (2.73, 3.33)	0.32 (-0.23, 0.88)
Difference in mean boost by with or without strain change	1.54 (0.73, 2.39)	1.45 (0.94, 1.96)	

Supplementary Table 4. Simulation study for the model for estimating the cumulative incidence of infection. Simulation value of a parameter is set to be the mean of the corresponding posterior distribution.

parameter		Simulation value	Mean estimate	Proportion covered (over 50)
Parameters related to 1) H1N1	$\boldsymbol{\psi_{11}}$: children before change point	0.2	0.2	0.98
	$\boldsymbol{\psi_{12}}$: adults before change point	0.06	0.06	0.84
	$\boldsymbol{\psi_{13}}$: older adults before change point	0.04	0.04	0.96
	$\boldsymbol{\psi_{11}}'$: children in after change point	0.49	0.49	0.94
	$\boldsymbol{\psi_{12}}'$: adults in after change point	0.09	0.09	1
	$\boldsymbol{\psi_{13}}'$: older adults after change point	0.07	0.09	0.94
Parameters related to 2) H3N2	$\boldsymbol{\psi_{21}}$: children	0.36	0.36	0.88
	$\boldsymbol{\psi_{22}}$: adults	0.27	0.28	0.98
	$\boldsymbol{\psi_{23}}$: older adults	0.3	0.32	0.92
	$\boldsymbol{\beta_1}$**:** protection of HAI titers	-0.58	-0.64	0.9
Parameters related to 3) H1N1	$\boldsymbol{\psi_{31}}$: children	0.8	0.88	0.92
	$\boldsymbol{\psi_{32}}$: adults	0.43	0.46	0.86
	$\boldsymbol{\psi_{33}}$: older adults	0.28	0.31	0.94
	$\boldsymbol{\beta_2}$**:** protection of HAI titers	-0.1	-0.1	1
Parameters related to 4) H3N2	$\boldsymbol{\psi_{41}}$: children	0.38	0.38	0.9
	$\boldsymbol{\psi_{42}}$: adults	0.18	0.19	0.96
	$\boldsymbol{\psi_{43}}$: older adults	0.2	0.2	0.98
	$\boldsymbol{\beta_3}$**:** protection of HAI titers	-0.91	-1.03	0.86
Parameters related to 5) H3N2	$\boldsymbol{\psi_{51}}$: children	1.09	1.3	0.94
	$\boldsymbol{\psi_{52}}$: adults	0.29	0.35	0.88
	$\boldsymbol{\psi_{53}}$: older adults	0.32	0.38	0.96
	$\boldsymbol{\beta_4}$**:** protection of HAI titers	-0.15	-0.15	1
Parameters related to 6) H3N2	$\boldsymbol{\psi_{61}}$: children	0.5	0.61	0.9
	$\boldsymbol{\psi_{62}}$: adults	0.34	0.39	0.88
	$\boldsymbol{\psi_{63}}$: older adults	0.16	0.2	0.96
	$\boldsymbol{\beta_5}$**:** protection of HAI titers	-0.83	-0.93	0.9
	$\boldsymbol{\alpha_{b11}}$**:** mean boosting for children	5.1	5.08	0.96

Boosting and waning for H1N1 with strain change (Epidemic 1)	α_{b12}: mean boosting for adults	0.33	0.33	0.98
	α_{w11}: mean waning for children	4.44	4.42	0.94
	α_{w12}: mean waning for adults	0.61	0.62	0.88
Boosting and waning for H1N1 without strain change (Epidemic 3 and 5)	α_{b21}: mean boosting for children	3.22	3.31	0.86
	α_{b22}: mean boosting for adults	0.04	0.05	1
	α_{w21}: mean waning for children	3.1	3.07	0.92
	α_{w22}: mean waning for adults	0.07	0.07	0.98
Boosting and waning for H3N2 with strain change (Epidemic 4)	α_{b31}: mean boosting for children	4.29	4.32	1
	α_{b32}: mean boosting for adults	0.24	0.26	0.92
	α_{w31}: mean waning for children	4.18	4.19	1
	α_{w32}: mean waning for adults	0.19	0.2	0.96
Boosting and waning for H3N2 without strain change (Epidemic 2 and 6)	α_{b41}: mean boosting for children	3.81	3.9	0.98
	α_{b42}: mean boosting for adults	0.03	0.08	0.96
	α_{w41}: mean waning for children	3.05	3.09	0.9
	α_{w42}: mean waning for adults	0.12	0.14	0.98
Parameter of measurement error	σ_1^2 : parameter for one side error for H1N1	2.12	2.14	1
	σ_2^2 : parameter for one side error for H3N2	1.57	1.58	1
	ϵ : probability that measurement went wrong so that the observed HAI titer was a random value in between 0 to 9	0.034	0.034	0.94

Supplementary Table 5. Summary of the variables in the models

Measurement model		Prior distribution
Unknown model parameter		
$\boldsymbol{\sigma_1^2}$: parameter for one side error for H1N1		$Uniform(0,100)$
$\boldsymbol{\sigma_2^2}$: parameter for one side error for H3N2		$Uniform(0,100)$
$\boldsymbol{\epsilon}$ **:** probability that measurement went wrong so that the observed HAI titer was a random value in between 0 to 9		$Uniform(0,1)$
**HAI titer dynamics model**		
Unknown model parameter		
Boosting and waning for H1N1 with strain change (Epidemic 1)	$\boldsymbol{\alpha_{b11}}$**:** mean boosting for children	$Uniform(0,100)$
	$\boldsymbol{\alpha_{b12}}$**:** mean boosting for adults	$Uniform(0,100)$
	$\boldsymbol{\alpha_{w11}}$**:** mean waning for children	$Uniform(0,100)$
	$\boldsymbol{\alpha_{w12}}$**:** mean waning for adults	$Uniform(0,100)$
Boosting and waning for H1N1 without strain change (Epidemic 3 and 5)	$\boldsymbol{\alpha_{b21}}$**:** mean boosting for children	$Uniform(0,100)$
	$\boldsymbol{\alpha_{b22}}$**:** mean boosting for adults	$Uniform(0,100)$
	$\boldsymbol{\alpha_{w21}}$**:** mean waning for children	$Uniform(0,100)$
	$\boldsymbol{\alpha_{w22}}$**:** mean waning for adults	$Uniform(0,100)$
Boosting and waning for H3N2 with strain change (Epidemic 4)	$\boldsymbol{\alpha_{b31}}$**:** mean boosting for children	$Uniform(0,100)$
	$\boldsymbol{\alpha_{b32}}$**:** mean boosting for adults	$Uniform(0,100)$
	$\boldsymbol{\alpha_{w31}}$**:** mean waning for children	$Uniform(0,100)$
	$\boldsymbol{\alpha_{w32}}$**:** mean waning for adults	$Uniform(0,100)$
Boosting and waning for H3N2 without strain change (Epidemic 2 and 6)	$\boldsymbol{\alpha_{b41}}$**:** mean boosting for children	$Uniform(0,100)$
	$\boldsymbol{\alpha_{b42}}$**:** mean boosting for adults	$Uniform(0,100)$
	$\boldsymbol{\alpha_{w41}}$**:** mean waning for children	$Uniform(0,100)$
	$\boldsymbol{\alpha_{w42}}$**:** mean waning for adults	$Uniform(0,100)$
Latent variables		

α_i: *I*ndividual boosting level for the HAI titers for an infection *i* for epidemic *j* and age group *k*		$Gamma(\alpha_{bjk}, 1)$
γ_i: individual yearly decay rate for the HAI titers for individual *i* for epidemic *j* and age group *k*		$Gamma(\alpha_{wjk}, 1)$
**Infection model**		
Unknown model parameter		
Scale parameters related to 1) H1N1, to define the probability of infection	$\boldsymbol{\psi_{11}}$: children before change point	$Uniform(0,100)$
	$\boldsymbol{\psi_{12}}$: adults before change point	$Uniform(0,100)$
	$\boldsymbol{\psi_{13}}$: older adults before change point	$Uniform(0,100)$
	$\boldsymbol{\psi_{11}}'$: children in after change point	$Uniform(0,100)$
	$\boldsymbol{\psi_{12}}'$: adults in after change point	$Uniform(0,100)$
	$\boldsymbol{\psi_{13}}'$: older adults after change point	$Uniform(0,100)$
Scale parameters related to 2) H3N2, to define the probability of infection	$\boldsymbol{\psi_{21}}$: children	$Uniform(0,100)$
	$\boldsymbol{\psi_{22}}$: adults	$Uniform(0,100)$
	$\boldsymbol{\psi_{23}}$: older adults	$Uniform(0,100)$
	$\boldsymbol{\beta_1}$**:** protection of HAI titers	$Normal(0,1)$
Scale parameters related to 3) H1N1, to define the probability of infection	$\boldsymbol{\psi_{31}}$: children	$Uniform(0,100)$
	$\boldsymbol{\psi_{32}}$: adults	$Uniform(0,100)$
	$\boldsymbol{\psi_{33}}$: older adults	$Uniform(0,100)$
	$\boldsymbol{\beta_2}$**:** protection of HAI titers	$Normal(0,1)$
Scale parameters related to 4) H3N2, to define the probability of infection	$\boldsymbol{\psi_{41}}$: children	$Uniform(0,100)$
	$\boldsymbol{\psi_{42}}$: adults	$Uniform(0,100)$
	$\boldsymbol{\psi_{43}}$: older adults	$Uniform(0,100)$
	$\boldsymbol{\beta_3}$**:** protection of HAI titers	$Normal(0,1)$
Scale parameters related to 5) H3N2, to define the probability of infection	$\boldsymbol{\psi_{51}}$: children	$Uniform(0,100)$
	$\boldsymbol{\psi_{52}}$: adults	$Uniform(0,100)$
	$\boldsymbol{\psi_{53}}$: older adults	$Uniform(0,100)$
	$\boldsymbol{\beta_4}$**:** protection of HAI titers	$Normal(0,1)$
Scale parameters related to 6) H3N2, to define the probability of infection	$\boldsymbol{\psi_{61}}$: children	$Uniform(0,100)$
	$\boldsymbol{\psi_{62}}$: adults	$Uniform(0,100)$
	$\boldsymbol{\psi_{63}}$: older adults	$Uniform(0,100)$
	$\boldsymbol{\beta_5}$**:** protection of HAI titers	$Normal(0,1)$
Latent variables		
$\boldsymbol{y_i}$: *I*ndividual infection status		$P(y_i = 1) = P(y_i = 0) = 0.5$
$\boldsymbol{t_i}$: *I*ndividual infection time		$Discrete\ Uniform(t_a, t_b)$, where t_a, t_b are the start and

	the end of follow-up of an epidemic
**Model for pre-epidemic HAI titer distribution**	
Model unknown parameter	
$\boldsymbol{p_k^{ij}}$**:** the probability an individual *i* in age group *j* with true pre-epidemics titer in *[k,k+1[. k=0.,1,..,9*	Dirichlet prior distribution with hyperparameter $\alpha_{baseline}$
Latent variable	
$\boldsymbol{x^{ij}}$: the true pre-epdiemics titer for individual *i* in epidemis *j*	$Uniform(0,10)$

Supplementary Note 1: Additional details of study design

Design

Data were collected from two community-based randomized controlled trials for evaluating direct and indirect benefits of influenza vaccination [1, 2]. In 2008–2009, 119 households were enrolled and one child 6-15 years old in each household was randomly allocated to receive either a single dose of trivalent-inactivated influenza vaccine (TIV) or saline placebo. Serum specimens were collected at enrollment to the study in November–December 2008, in April 2009, and in August–October 2009 from each household member [1].

In a larger trial in 2009–2010, 796 households including 83 of the 119 households from the previous study were enrolled. One child 6–17 years old per household was randomly allocated to receive either a single dose of TIV or saline placebo [2]. Serum specimens were collected at enrollment in August-December 2009 and at the end of the study in August-December 2010. We also collected serum specimens from 25% of the household plus from all 83 households that continued from the prior study in April-May 2010. In both studies, we collected one additional serum specimen 1 month after vaccination from children who received TIV or placebo. After the trial, participants were followed up over multiple years, with collection of serum specimens in October-December each year. Those households provided serum specimens in April-May 2010 continued to provided serum in April-May each year [3].

Supplementary Note 2: Model for estimating infection risk of influenza from serology data

One traditional method to estimate risk of influenza virus infection in epidemic is using serology [4]. Paired sera are collected before and after epidemic, and 4-fold or greater rise in antibody titers measured by an assay, such as haemagglutination inhibition (HAI) assay, is considered as evidence of infections. However, in tropical or sub-tropical area such as Hong Kong, the paired sera may not neatly bracket the epidemics due to the unpredictable timing of influenza epidemic [5]. Therefore, some infections could be missed by using 4-fold criterion as an indicator of infections due to the followings:

1) If the first serum is collected after the beginning of epidemic, infections could occur before the collection. Hence those individuals may need to be excluded in analysis.

2) Waning in antibody could cause missing infections if the duration between two serums is too long, so that 4-fold or greater rise disappear due to waning in antibody.

3) Measurement error in the serum data causes misclassification in using 4-fold criterion as an indicator of infection.

Here, we aim to develop a comprehensive model that couples surveillance data and serology data to estimate the infection risk with addressing such issues. In that model, the unobserved HAI titer trajectory for each individual is reconstructed to infer the infection status, instead of using 4-fold criterion. Also, we identify factors affecting the infection risk.

Antibody titer measurement and study design

For each serum sample, antibody titer levels are measured by haemagglutination inhibition assay (HAI). The following two-fold dilutions are used: 1:10, 1:20, 1:40, 1:80, 1:160, 1:320, 1:640, 1:1280, 1:2560. Hereafter, they are translated onto a $\log_2$ scale, therefore 1:10 is 1, 1:20 is 2 and so on. Those with <1:10 is assigned to 0.

For our study, sera were collected from April 2009 to November 2013, in half-year intervals. However, there were missing data due to the followings:

1) Only one-fifth of participants provided sera on April 2009.

2) Two-third of participants provided sera yearly, on November each year.

3) Participants could leave the study on November each year.

Our aim is to develop statistical models to estimating the infection risk for 6 influenza A epidemics in the study period, accounting for abovementioned issues, listed below:

1) A(H1N1)pdm09 from July 5, 2009 to January 16, 2010

2) A(H3N2) from August 1 to October 16, 2010

3) A(H1N1)pdm09 from January 9 to February 26, 2011

4) A(H3N2) from March 11 to June 23, 2012

5) A(H1N1) pdm09 from February 17 to April 20, 2013

6) A(H3N2) from July 14 to October 12, 2013

Model overview

We build a 5-level Bayesian hierarchical model to integrate serology and surveillance data to estimate infection risk for each epidemic. For the measured pre-epidemic (baseline) titer AT_b^* and non-baseline titer AT^*, 'true' baseline titer AT_b and non-baseline titer AT, individual boosting and waning parameters μ_{bw}, infection status y, infection time t_I, age group a, surveillance data P and parameter vector θ, we have

$P(AT_b^*, AT^* | AT_b, AT, \theta) * P(AT | \mu_{bw}, y, t_I, AT_b, \theta, a) * P(y, t_I, | AT_b, \theta, a, P) *$
$P(AT_b | \theta, a) * P(\theta)$ (Eq. 1)

Here, $P(AT_b^*, AT^* | AT_b, AT, \theta)$ is the measurement model, $P(AT | \mu_{bw}, y, t_I, AT_b, \theta, a)$ is the HAI titer dynamics model, $P(y, t_I | AT_b, \theta, a, P)$ is the infection model, and $P(AT_b | \theta, a)$ is the baseline 'true' titer distribution. $P(\theta)$ is the prior distribution of model parameter. Parameters and latent variables in

the model were summarized in Supplementary Table 5. The details of each level are in the following sections.

Measurement model

As defined in the main text, we model the underlying titer on a continuous scale, so that a true titer between any two dilutions is measured as the lower of the two dilutions when there is no measurement error. For example, a true titer of 1.7 would be measured as 1, since the dilutions performed at 1, 2, 3, 4 etc. Denote AT and AT^* as the "true" and observed titers respectively. Denote $[x]$ as the integer part of x. We use the approach in Cauchemez et al. to model the probability of 2-fold error on the left or right [4]. Then, we denote that the 1-sided probability of 2-fold error on the left is:

$$P(AT^* = [AT] - 1|AT) = \frac{[AT] + 1 - AT}{2 * \exp(\sigma)} \text{ (Eq. 2)}$$

And the 1-sided probability of 2-fold error on the right is:

$$P(AT^* = [AT] + 1|AT) = \frac{AT - [AT]}{2 * \exp(\sigma)} \text{ (Eq. 3)}$$

Where σ is the parameter to quantify the 1-sided probability of two-fold error, larger value of σ implies smaller the two-fold error.

Hence the probability of no error is:

$$P(AT^* = [AT]|AT) = \begin{cases} 1 - P(AT^* = [AT] - 1|AT) - P(AT^* = [AT] + 1|AT) \; if \; AT \geq 1 \\ 1 - P(AT^* = [AT] + 1|AT) \; if \; AT < 1 \end{cases} \text{ (Eq. 4)}$$

We also denote ϵ the probability that measurement goes wrong and the observed titer is a random value in between 0 to 9.

We use two different parameters for the error distribution to account for the potential difference in measurement error in H1N1 and H3N2 assay.

HAI titer dynamics model

When an individual is infected at a given day in an epidemic, his/her titer receives a boost. Denote i as an infection in an epidemic for an individual. We

assume the magnitude of such boost follows a Gamma distribution, and the boosting takes 14 days [5, 6]:

$$AT_i(t_i + 14) = AT_i(t_i) + \alpha_i \text{ (Eq. 5)}$$

where infection time is t_i and α_i is the individual boosting level for the HAI titers for an infection *i.* We assume the individual boosting level follows a Gamma distribution:

$$\alpha_i \sim Gamma(\alpha_b, 1) \text{ (Eq. 6)}$$

α_b is the parameter for the boosting distribution.

We also assume such boosting is temporary and does decay over time [7]. Titer level after *u* days is:

$$AT_i(t_i + u) = AT_i(t_i) * \exp\left(-\gamma_i * \frac{u}{365}\right) \text{ (Eq. 7)}$$

where $u > 14$, γ_i is individual yearly decay rate for the HAI titers for individual *i.* We assume γ_i follows a Gamma distribution:

$$\gamma_i \sim Gamma(\alpha_w, 1) \text{ (Eq. 8)}$$

where α_w is the parameter for the waning distribution. We allowed these boosting and waning distribution to be different by age groups (children and adults), subtype (H1N1 and H3N2) and epidemics with or without strain change. In our model, the scale parameter for the Gamma distribution is fixed to be 1, since simulation suggested that the shape and scale parameters could not converge for the current sample size. However, this assumption can be relaxed if sample size is sufficient.

In total there were 8 sets of boosting and waning distribution. We group these boosting and waning parameters as a parameter vector μ_{bw}.

Infection model

We use the influenza proxy (denoted as *P*) to approximate influenza activity [8]. Details are in the surveillance data section in the main text. The hazard of infection at time *t* is

$$\lambda(t|a, AT_b) = \psi_a * P_t * \exp(-\beta * AT_b) \text{ (Eq. 9)}$$

where ψ_a is the scaling factor for the risk of infection for strain *j* the age group a (1: children ≤17, 2: adults 18-50, 3: older adults ≥51), P_t is the influenza activity proxy at time *t* based on local surveillance data, and β is the effect associated with the pre-epidemic titers. β is set to be 0 in epidemic 1, since almost everyone is naïve (pre-pandemic HAI titer < 1:10) in the pandemic H1N1 outbreak in 2009.

The probability of infection at time t, is:

$$\text{P}(y = 1, t) = \{\exp[-\sum_{u=1}^{t-1} \lambda(u|a, AT_b)]\}\{1 - \exp[-\lambda(t|a, AT_b)]\} \text{ (Eq. 10)}$$

Denote the duration of the epidemic as T. For non-infection, it is:

$$\text{P}(y = 0) = \exp[-\sum_{u=1}^{T} \lambda(u|a, AT_b)] \text{ (Eq. 11)}$$

Pre-epidemic HAI titer distribution

For each epidemic, we assume the integer part of the 'true' pre-epidemic titer follows a multinomial distribution with 10 levels (0 to 9), and the non-integer part follows a uniform distribution. The density of this distribution:

$$f(x) = p_{[x]}, x \in [0,10) \text{ (Eq. 12)}$$

These distributions are estimated separately for children (≤17) and adults (≥18) for each epidemic.

Supplementary Note 3: Details of inference of models

Estimation is performed in a Bayesian framework. Data augmentation was used to sample the unobserved variables.

Priors

For the parameters that only take positive value, we use a vague Uniform(0,100) prior. For the parameters about protection associated with HAI titer, we use a Normal(0,1) prior. It is because in some situations that all individuals with HAI titer are imputed to be uninfected, this parameter would be estimated to be a very small value (its theoretic value should be -Inf to reduce the probability of infection for such group of individuals to be 0).

We use a Dirichlet prior distribution with hyperparameter $\alpha_{baseline}$ for a multinomial distribution.

Algorithm

We develop a reversible jump MCMC to reconstruct the infection status and infection time, individual boosting and waning parameters.

At each MCMC step k, we have three main steps:

1) Update the model parameters

We update the model parameters θ by using random walk Metropolis-Hastings algorithm [9]. The step size of the proposal is adjusted to have acceptance probability for 20-30%.

The multinomial distributions for the pre-epidemic HAI titer distribution are updated using Gibbs sampler as follows: Suppose in step k, the number of individuals with the integer part of the true baseline HAI titer levels u, is n_u. Denote $n = \{n_u, u = 0,1,..,9\}$. Then the posterior distribution of $\delta = \{\delta_u, u = 0,1,..,9\}$ is $Dirichlet(n + \alpha_{baseline})$.

Hyperparameters for the multinomial distributions are updated by Metropolis-Hastings algorithm.

2) Update individual parameters

In this step, for each individual, the following individual parameters are updated, with the following proposal distributions:

a) Pre-epidemic HAI titer AT_b: The proposal distribution is the pre-epidemic HAI titer distribution at step *k.*

b) Infection time: Infection time is proposed from the set of potential infection time with equal probability. Potential infection time is defined as from the start of follow-up (time of collection of the first serum) to the end of follow-up (time of collection of the last serum) for an epidemic.

c) Individual boosting parameter α_i: The proposal distribution is the boosting distribution $Gamma(\alpha_b, 1)$ at step *k.*

d) Individual waning parameter γ_i: The proposal distribution is the waning distribution $Gamma(\gamma_w, 1)$ at step *k.*

In this update, the infection status for each individual is fixed. Therefore, the update is using Metropolis-Hastings algorithm based on the joint likelihood of parameters and the augmented data.

3) Reversible jump MCMC to update infection status for each individual.

This step is to reconstruct the infection status for each individual. Due to the limitation of using the traditional 4-fold criterion to identify infections mentioned in the introduction in the main text, we use Bayesian approach to probabilistically identify infections. Reversible jump is needed, since when an individual is imputed to be infected from uninfected in the MCMC step *k-1*, there are additional parameter including the infection time and the parameter of individual boost. Hence, the number of latent parameters is increased. This number is decreased when an individual is imputed to be uninfected from infected in the MCMC step *k-1*.

We start from randomly select 20% of individuals as infection with infection time randomly draw from the study period. In each MCMC step, we conduct the update as follows:

A) Add an infection. We randomly select an individual imputed to be uninfected. Then we draw candidates for infection time t, individual boosting parameter α_i, individual waning parameter γ_i and baseline antibody titers AT_b. The proposal distributions are the same as a) to d) in updating individual parameters.

B) Remove an infection. We randomly select an individual imputed to be infected. Then we draw candidate for individual waning parameter γ_i and baseline antibody titers AT_b. The proposal distributions are the same as A).

Suppose in step *k,* there is X imputed infected individual and Y imputed uninfected individual. Hence the proposal ratio reversible jump MCMC moves for A) is

$$\frac{Y * P(AT_b) * P(\gamma_i)}{(X+1) * P(t') * P(\alpha_i') * P(\gamma_i') * P(AT_b{}')} \text{ (Eq. 13)}$$

for B) is

$$\frac{X * P(t) * P(\alpha_i) * P(\gamma_i) * P(AT_b)}{(Y+1) * P(AT_b') * P(\gamma_i')} \text{ (Eq. 14)}$$

The algorithm runs for 200000 iterations after a burn-in of 100000 iterations, with a thining of 10. Converge is visually assessed. One run takes about 15 hours on a typical desktop.

Supplementary Note 4: Association between infection status in different epidemics

Among 2353 individuals in our analysis, 1853 of them experienced more than one epidemic, and 937 of them experienced all six epidemics. Hence, based on the individuals participated in more than one epidemic, we explore the association between infection status in different epidemics, by fitting a logistic regression of infection status for an epidemic A on the infection status for a prior epidemic B. Protection is defined as one minus odds ratio for individuals with previous infection verses individuals without previous infection. In our study, homosubtypic protection was quantified by the association between infection status in epidemics 1, 3 and 5 (H1N1) or in epidemic 2, 4 and 6 (H3N2). Other pairs were heterosubtypic protections (Table S2). The results are plotted in Figure 3 and Figure S2. It shows that there is no heterosubtypic protection from previous infection. For homosubtypic protection, it is only available for H1N1 but not H3N2.

We further explore if the identified homosubtypic protection could be explained by pre-epidemic HAI titers. Therefore we included the pre-epidemic HAI titer in the model, and found that the identified homosubtypic protection is no longer significant, suggesting that the homosubtypic protection from previous infection could be explained by the boosting of HAI titer after infection, resulting in higher level of pre-epidemic HAI titer.

We follow the approach in Salje et al. to account for sampling uncertainty [10]. We do 100 bootstraps to conduct logistic regression for each of 100 augmented datasets. Then we present the mean, 2.5 and 97.5 quantiles for those 10000 (100 augmented data times 100 bootstrap samples for each augmented data) curves.

Supplementary Note 5: Model validation and adequacy

To assess the performance of our estimation procedure, we perform a simulation study. We first simulate 50 datasets with a structure identical to that of the observed data (in terms of age, time of serum drawing and the availability of serum in each round) and simulate the infection status with parameters set to be their posterior mean. Then we applied our estimation procedure to the simulated data sets and tested if the parameters could be estimated. The result of simulation was summarized in Table S4. We found that in general the true value was in the credible interval in about 84% to 100% of times for the 45 parameters for the infection risk, boosting and waning parameter, and parameter of measurement error. It suggested that our estimation procedure would be able to provide reasonable estimates of the parameter.

We also checked our model adequacy by comparing the distribution of titer changes for each age group and each epidemic in observed data and the 1000 simulated epidemics. Data were simulated with a structure identical to that of the observed data (in terms of age, time of serum drawing and the availability of serum in each round), with parameters randomly drawn from their posterior distribution. The results are summarized in Figure S4. The distribution of titer changes from the 1000 simulated epidemics are consistent with the observed one in all age groups and epidemics, suggested that the model provide reasonable fit to the data.

Supplementary References

1. Cowling BJ, Ng S, Ma ES, Cheng CK, Wai W, Fang VJ, et al. Protective efficacy of seasonal influenza vaccination against seasonal and pandemic influenza virus infection during 2009 in Hong Kong. Clin Infect Dis. 2010;51(12):1370-9. doi: 10.1086/657311. PubMed PMID: 21067351.
2. Cowling BJ, Ng S, Ma ES, Fang VJ, So HC, Wai W, et al. Protective efficacy against pandemic influenza of seasonal influenza vaccination in children in Hong Kong: a randomized controlled trial. Clin Infect Dis. 2012;55(5):695-702. doi: 10.1093/cid/cis518. PubMed PMID: 22670050.
3. Cowling BJ, Perera RA, Fang VJ, Chan KH, Wai W, So HC, et al. Incidence of influenza virus infections in children in Hong Kong in a 3-year randomized placebo-controlled vaccine study, 2009-2012. Clin Infect Dis. 2014;59(4):517-24. doi: 10.1093/cid/ciu356. PubMed PMID: 24825868.
4. Cauchemez S, Horby P, Fox A, Mai le Q, Thanh le T, Thai PQ, et al. Influenza infection rates, measurement errors and the interpretation of paired serology. PLoS Pathog. 2012;8(12):e1003061. doi: 10.1371/journal.ppat.1003061. PubMed PMID: 23271967; PubMed Central PMCID: PMC3521724.
5. Tsang TK, Fang VJ, Perera RA, Ip DK, Leung GM, Peiris JS, et al. Interpreting Seroepidemiologic Studies of Influenza in a Context of Nonbracketing Sera. Epidemiology. 2016;27(1):152-8. doi: 10.1097/EDE.0000000000000408. PubMed PMID: 26427725; PubMed Central PMCID: PMCPMC4825848.
6. Veguilla V, Hancock K, Schiffer J, Gargiullo P, Lu X, Aranio D, et al. Sensitivity and specificity of serologic assays for detection of human infection with 2009 pandemic H1N1 virus in U.S. populations. Journal of clinical microbiology. 2011;49(6):2210-5. doi: 10.1128/JCM.00229-11. PubMed PMID: 21471339; PubMed Central PMCID: PMC3122722.
7. Zhao X, Ning Y, Chen MI, Cook AR. Individual and Population Trajectories of Influenza Antibody Titers Over Multiple Seasons in a Tropical Country. Am J Epidemiol. 2018;187(1):135-43. doi: 10.1093/aje/kwx201. PubMed PMID: 29309522; PubMed Central PMCID: PMCPMC5860523.
8. Wong JY, Kelly H, Ip DK, Wu JT, Leung GM, Cowling BJ. Case fatality risk of influenza A (H1N1pdm09): a systematic review. Epidemiology. 2013;24(6):830-

41. Epub 2013/09/21. doi: 10.1097/EDE.0b013e3182a67448. PubMed PMID: 24045719; PubMed Central PMCID: PMCPMC3809029.
9. Gilks WR, Richardson S, Spiegelhalter D. Markov Chain Monte Carlo in Practice. London: Chapman & Hall; 1996.
10. Salje H, Cummings DAT, Rodriguez-Barraquer I, Katzelnick LC, Lessler J, Klungthong C, et al. Reconstruction of antibody dynamics and infection histories to evaluate dengue risk. Nature. 2018;557(7707):719-23. doi: 10.1038/s41586-018-0157-4. PubMed PMID: 29795354; PubMed Central PMCID: PMCPMC6064976.
